# Bright light therapy for post-stroke sleep disturbances: a systematic review and meta-analysis

**DOI:** 10.3389/fneur.2026.1875347

**Published:** 2026-09-07

**Authors:** Xiaolan Tang, Qiuping Gu, Yang Zhan, Xiaomei Sun, Fengqin Wang, Luchen Pan, Hua Zhao

**Affiliations:** Nursing Department, The Second Affiliated Hospital of Zhejiang University School of Medicine, Hangzhou, China

**Keywords:** bright light therapy, insomnia, meta-analysis, sleep disturbances, stroke

## Abstract

**Objective:**

Sleep disturbances are common after stroke and may adversely affect neurological recovery. Although post-stroke sleep disturbances encompass insomnia symptoms, circadian rhythm sleep–wake disturbances, and sleep-disordered breathing, research has focused predominantly on obstructive sleep apnea. This review evaluated the effects of bright light therapy (BLT), a non-pharmacological intervention that may influence circadian regulation, on sleep outcomes after stroke.

**Methods:**

Ten electronic databases covering English-and Chinese-language literature were searched from inception to April 9, 2026. Randomized controlled trials involving adults with stroke that evaluated validated subjective or objective sleep outcomes were eligible. Data were pooled using random-effects models when studies were sufficiently comparable clinically and methodologically. Risk of bias was assessed using the Cochrane Risk of Bias 2 tool. The primary outcomes were wake after sleep onset (WASO) and subjective sleep quality. The protocol was registered in PROSPERO (CRD420251111617).

**Results:**

Five randomized controlled trials involving 343 participants were included. Three trials involving 181 participants contributed to the WASO analysis; the pooled estimate was not statistically significant (MD = −39.17 min, 95% CI −115.34 to 36.99; P = 0.31), and heterogeneity was substantial (I^2^ = 96%). Four trials involving 300 participants contributed to the subjective sleep quality analysis; the pooled estimate favored BLT (SMD = −0.85, 95% CI −1.38 to −0.32; P = 0.002), although heterogeneity was substantial (I^2^ = 78%). After exclusion of Song Chang-yu, the pooled effect remained statistically significant but was attenuated, and heterogeneity decreased to 0% (SMD = −0.55, 95% CI −0.83 to −0.28).

**Conclusion:**

BLT may improve subjective sleep quality after stroke; however, evidence for objective sleep continuity remains uncertain because of the small evidence base and substantial heterogeneity. Larger, methodologically rigorous trials using standardized light parameters, standardized outcome measures, and direct circadian assessments are needed to clarify the clinical effects of BLT and identify optimal treatment protocols.

**Systematic Review Registration:**

https://www.crd.york.ac.uk/PROSPERO/view/CRD420251111617, identifier CRD420251111617.

## 1 Introduction

Stroke remains one of the leading causes of death and long-term disability worldwide (1). Sleep disturbances are among the most common post-stroke complications, and their potential impact on rehabilitation and long-term outcomes has received increasing attention as stroke survival has improved (2). Previous meta-analyses have estimated the overall prevalence of post-stroke insomnia at approximately 48.37% (3). Furthermore, evidence suggests that prevalence estimates based on non-diagnostic screening tools are higher than those based on diagnostic criteria, indicating that symptom-based assessments may capture a broader range of sleep complaints and that post-stroke insomnia remains under-recognized in clinical practice (4).

Growing evidence indicates that post-stroke sleep disturbances are associated with adverse clinical outcomes, including poorer functional recovery, cognitive decline, and unfavorable neurological outcomes (5, 6). Prospective cohort studies further suggest that sleep–wake disturbances after stroke are associated with an increased risk of subsequent cardio-cerebrovascular events and all-cause mortality, with each unit increase in a sleep burden index corresponding to an approximately 80% higher risk of these outcomes (7). Post-stroke sleep disturbances are multifactorial and may reflect lesion-related disruption of sleep-wake regulatory networks, environmental changes during hospitalization, and alterations in circadian regulation (6, 8, 9). Although post-stroke sleep disturbances encompass insomnia symptoms, circadian rhythm sleep-wake disturbances, and sleep-disordered breathing, research has focused predominantly on obstructive sleep apnea (10). By contrast, insomnia symptoms and impaired sleep continuity remain under-recognized during stroke rehabilitation.

Current expert consensus emphasizes safe and feasible non-pharmacological approaches for the management of post-stroke sleep disturbances (11). Bright light therapy is a non-pharmacological intervention that delivers controlled light exposure through dedicated devices or environmental lighting systems. Through its effects on the circadian system, light exposure can influence sleep-wake regulation, providing a biological rationale for the use of bright light therapy in post-stroke sleep disturbances (12, 13). Bright light therapy can be delivered as full-spectrum or bright white light at illuminance levels above those typically encountered indoors, as well as wavelength-specific or blue-enriched light intended to enhance non-visual photic effects (14). Importantly, neural and circadian responses to light are strongly dependent on exposure timing and may exhibit threshold effects rather than a simple linear dose-response relationship, which may partly account for the heterogeneity of light-therapy protocols used in clinical and rehabilitation settings (15).

Several randomized controlled trials have evaluated bright light therapy for sleep disturbances after stroke, including studies using objective sleep parameters derived from actigraphy (16, 17). However, intervention protocols, outcome measures, and findings vary considerably, and the available evidence has not been quantitatively synthesized. Therefore, we conducted this systematic review and meta-analysis to evaluate the effects of bright light therapy on sleep quality and sleep continuity after stroke and to examine the extent to which the available evidence supports a potential role for circadian regulation.

## 2 Materials and methods

### 2.1 Protocol and registration

This systematic review and meta-analysis was registered in PROSPERO (CRD420251111617). The review was conducted and reported in accordance with the Preferred Reporting Items for Systematic Reviews and Meta-Analyses (PRISMA) 2020 statement (18) and followed the methodological guidance provided in the Cochrane Handbook for Systematic Reviews of Interventions (19). A comprehensive literature search was conducted in CNKI, Wanfang Data, VIP Database, SinoMed, PubMed, Web of Science, Embase, the Cochrane Library, Scopus, and EBSCOhost from database inception to April 9, 2026, with no restriction on publication date. Detailed search strategies for each database are provided in the Supplementary Appendix. All retrieved records were imported into EndNote (version 21.5), and duplicate records were removed. Two reviewers independently screened titles and abstracts to identify potentially eligible studies. Full-text reports were then assessed against the predefined eligibility criteria. Disagreements regarding study selection were resolved through discussion and, when necessary, consultation with a third reviewer.

### 2.2 Study selection

#### 2.2.1 Eligibility criteria

Studies were eligible if they met all of the following criteria: 1) participants were adults (≥18 years) with ischemic or hemorrhagic stroke confirmed by neuroimaging and/or medical records; 2) post-stroke sleep disturbances were assessed using validated subjective questionnaires and/or objective sleep measures, including insomnia symptom scales, sleep efficiency, wake after sleep onset, or actigraphy-derived sleep parameters; 3) the intervention involved therapeutic light exposure intended to improve sleep or sleep- wake regulation; 4) the study used a randomized controlled design with at least two study groups; and 5) the study was available as a full-text peer-reviewed journal article, academic thesis, or dissertation in English or Chinese and provided sufficient methodological detail and extractable outcome data.

#### 2.2.2 Exclusion criteria

Studies were excluded if they: 1) did not assess sleep using validated subjective or objective measures; 2) did not report sleep-related outcomes; 3) did not clearly confirm a diagnosis of stroke or provide separate data for participants with stroke; 4) evaluated an intervention unrelated to sleep; 5) did not involve therapeutic light exposure; or 6) were non-original publications, including reviews, protocols, conference abstracts, editorials, or commentaries.

### 2.3 Data extraction and reporting

#### 2.3.1 Data collection process

Two reviewers independently extracted data from the included studies and, where available, their Supplementary materials. The extracted light-intervention characteristics included population, setting, timing of exposure, illuminance, duration per session, number of sessions, total intervention period, light type or device, distance from the light source, and comparator. Spectral characteristics, including correlated color temperature, wavelength, and spectral power distribution, were recorded when reported.

To facilitate descriptive comparison across light-intervention protocols, nominal luminous exposure per session was calculated using the standard relationship between illuminance and exposure duration:


$$\begin{array}{l} H_{v}=Ev\times t \end{array}$$

where *H*_*v*_ represents luminous exposure, *Ev* is the reported illuminance in lux, and *t* is the duration of a treatment session in h. When session duration was reported in min, the value was divided by 60 before calculation. Luminous exposure per session was expressed in lux-h (lx·h) (20). When a study reported a fixed planned number of treatment sessions, cumulative nominal photopic exposure was calculated by multiplying the per-session luminous exposure by the total number of planned sessions.

Light-related parameters, including illuminance, exposure duration, session frequency, intervention duration, and light source characteristics, were extracted from each study. Melanopic metrics, including melanopic equivalent daylight illuminance (melanopic EDI) and melanopic irradiance, were not available in the included trials and therefore could not be quantitatively synthesized. The calculated lux-h values represent nominal photopic exposure and do not incorporate spectral composition or melanopsin-weighted biological effects (21).

### 2.4 Outcome data

Sleep-related outcomes included subjective sleep quality, wake after sleep onset (WASO), total sleep time (TST), sleep efficiency (SE), and daytime sleepiness. Subjective sleep quality and WASO were prespecified as primary outcomes because they reflect patient-perceived sleep impairment and objectively assessed sleep continuity, respectively. Other sleep parameters, including sleep efficiency, total sleep time, and daytime sleepiness, were considered secondary outcomes because they provide complementary information on sleep duration, sleep continuity, and daytime consequences. Circadian-related outcomes and biomarkers were extracted when available.

### 2.5 Risk of bias for individual studies

Risk of bias in each included randomized controlled trial was independently assessed by two reviewers using the revised Cochrane risk-of-bias tool for randomized trials (RoB 2) (22). Disagreements were resolved through discussion and, when necessary, consultation with a third reviewer. The assessment covered five domains: bias arising from the randomization process, bias due to deviations from intended interventions, bias due to missing outcome data, bias in measurement of the outcome, and bias in selection of the reported result. Overall risk-of-bias judgments were determined using the standard RoB 2 algorithm based on the domain-level judgments.

### 2.6 Data synthesis

Quantitative synthesis was undertaken when at least two studies reported sufficiently comparable post-intervention data with extractable variance estimates. Outcomes that did not meet these criteria were summarized narratively. Post-intervention scores were used in the primary analyses because change-from-baseline data were not consistently reported. Mean differences (MDs) were calculated when studies used the same measurement scale, whereas standardized mean differences (SMDs) were used when different validated instruments assessed the same construct. Outcomes with variance data that could not be reliably derived were excluded from quantitative synthesis and were described narratively. All meta-analyses used a random-effects model. Statistical heterogeneity was assessed using the *I*^2^ statistic, with values of approximately 25%, 50%, and 75% interpreted as low, moderate, and high heterogeneity, respectively. Analyses were conducted using Review Manager version 5.4.1.

### 2.7 Handling of the multi-arm trial

In the three-arm trial by Wang et al. (23) both the warm-light group (3000 K, 500 lux) and the cold-light group (6500 K, 500 lux) met the eligibility criteria for inclusion as structured morning light intervention. For quantitative synthesis, the two intervention arms were combined into a single light-intervention group, and the shared standard-lighting control group (5,000 K, 500 lux) was included only once to avoid double counting. The combined sample size was obtained by summing the sample sizes of the two intervention arms. The combined mean was calculated as a sample-size-weighted mean, and the pooled standard deviation was derived using the standard formula for combining groups recommended in the Cochrane Handbook (19).

## 3 Results

### 3.1 Study selection

**Figure 1:**
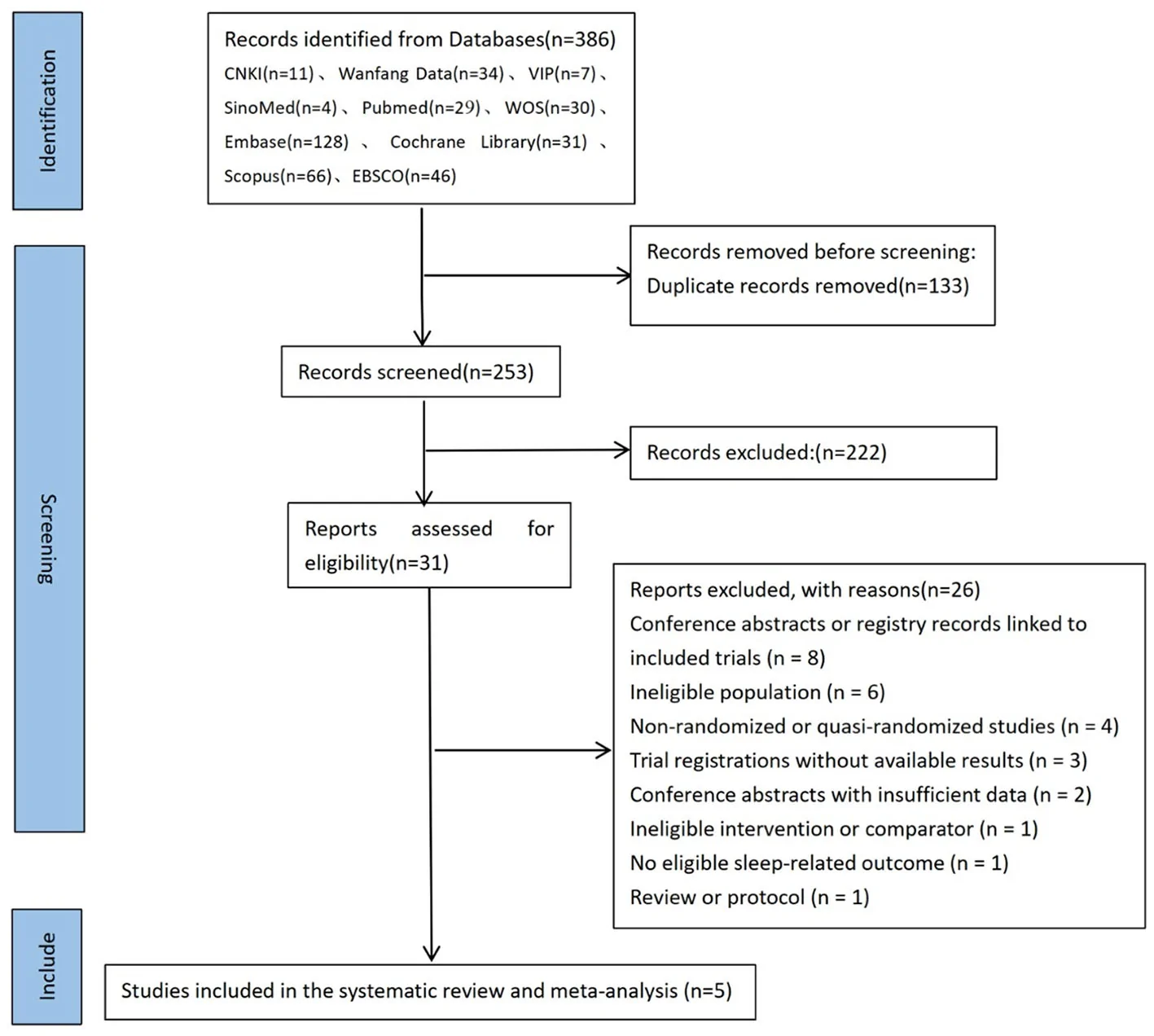
PRISMA flow diagram of the study selection process.

Figure 1 presents the PRISMA flow diagram for study identification, screening, and inclusion. A total of 386 records were identified through searches of ten electronic databases. After removal of 133 duplicate records, 253 records remained for title and abstract screening. Of these, 222 were excluded, and 31 full-text articles were assessed for eligibility. Twenty-six full-text reports were subsequently excluded for the reasons shown in Figure 1. Ultimately, five randomized controlled trials met the eligibility criteria and were included in the review.

### 3.2 Study characteristics

#### 3.2.1 Participants

Table 1 summarizes the characteristics of the five randomized controlled trials, published between 2021 and 2025. Together, the trials included 343 participants with post-stroke sleep disturbances, including 178 allocated to light-intervention groups and 165 to comparator groups. Four trials were conducted in inpatient rehabilitation or neurology settings, whereas Xiao et al. (24) evaluated a home-based intervention after recruiting participants from a stroke clinic. Mean age across studies ranged from 62.6 to 74.3 years, and the proportion of women ranged from 28.0% to 61.9%.

**Table 1:** Characteristics of the included randomized controlled trials and light-therapy interventions.

Study	Population and setting	Sample characteristics	Light exposure parameters				Photopic exposure (lx·h), session; cumulative	Light source and spectrum	Comparator
			Timing	Illuminance (lux)	Duration (min/session)	Sessions/period			
Kim et al. 2022 (16)	Early rehabilitation; ischemic or hemorrhagic stroke; inpatient rehabilitation, South Korea	56 (27/29); age 66.1 ± 13.7; female 43.0%	Shortly after awakening, preferably 07:00–08:00	10,000	30	14 sessions; 2 weeks	5,000; 70,000	Bright white light, Philips EnergyUp; 50–75 cm	Filtered placebo light, < 50 lux
Weeks et al. 2024 (17)	Acute rehabilitation; ischemic or hemorrhagic stroke; inpatient rehabilitation, USA	43 (21/22); age 67.3 ± 14.4; female 50.0%	08:00–08:30	400^*^	25	≥5 sessions; varied with length of stay	167; NR^*^	Blue LED, Philips goLITE BLU; 70 cm	Dim-red placebo light
Wang et al. 2025 (23)	Recovery phase, 1–3 months; ischemic stroke; inpatient, China	48 (16/16/16); age 62.6 ± 6.3; female 28.0%	08:30–09:30	500	60	20 sessions; 4 weeks	500; 10,000	Ceiling-mounted LED; 3,000 K, 5,000 K, or 6,500 K	Standard 5,000 K lighting
Xiao et al. 2021 (24)	Approximately 1 month post-stroke; ischemic stroke; home-based intervention, China	112 (56/56); age 64.1 ± 10.7; female 50.9%	08:00–09:00	10,000	30	42 sessions; 6 weeks	5,000; 210,000	White LED; reported wavelength 450–460 nm; 60 cm	Escitalopram alone
Song, 2021 (25)†	Stable phase; ischemic stroke; neurology ward, China	84 (42/42); age 74.3 ± 10.3; female 61.9%	Morning, shortly after awakening	10,000	30	7 sessions; 7 days	5,000; 35,000	Wearable blue–green light device; 450 nm	Routine care

Nominal photopic luminous exposure per session was calculated as illuminance multiplied by exposure duration in h. Cumulative nominal exposure was calculated as per-session luminous exposure multiplied by the planned number of sessions.

^*^The illuminance value for Weeks et al. was obtained from the supplementary spectral specifications. Cumulative exposure was not calculated because the number of sessions varied according to length of stay. NR, not reported or not calculable.

^†^Song CY (2021) was a master's dissertation rather than a peer-reviewed journal article.

### 3.3 Interventions

All five trials delivered light exposure in the morning or shortly after awakening. Intervention protocols varied in illuminance, exposure duration, intervention period, spectral characteristics, and light-delivery device. Illuminance ranged from 400 to 10,000 lx, session duration from 25 to 60 min, and intervention periods from five days to six weeks.

Three trials administered light at 10,000 lx for 30 min per session: Kim et al. (16) used bright white light for 14 sessions over two weeks, Xiao et al. (24) used white LED light for 42 sessions over six weeks, and Song (25) used a wearable blue–green light device for seven sessions over seven days. Weeks et al. (17) administered blue LED light at 400 lux for 25 min per session, with at least five sessions and the total number determined by length of stay. Wang et al. (23) administered ceiling-mounted LED lighting at 500 lx for 60 min per session over 20 sessions, with warm-light and cold-light intervention arms and a standard-lighting comparator. Where calculable, nominal photopic exposure ranged from 167 to 5,000 lx·h per session, and cumulative exposure ranged from 10,000 to 210,000 lx·h. Comparator conditions included filtered or dim-red placebo light, standard 5,000 K lighting, escitalopram alone, and routine care.

### 3.4 Risk of bias assessment of included randomized controlled trials

**Figure 2:**
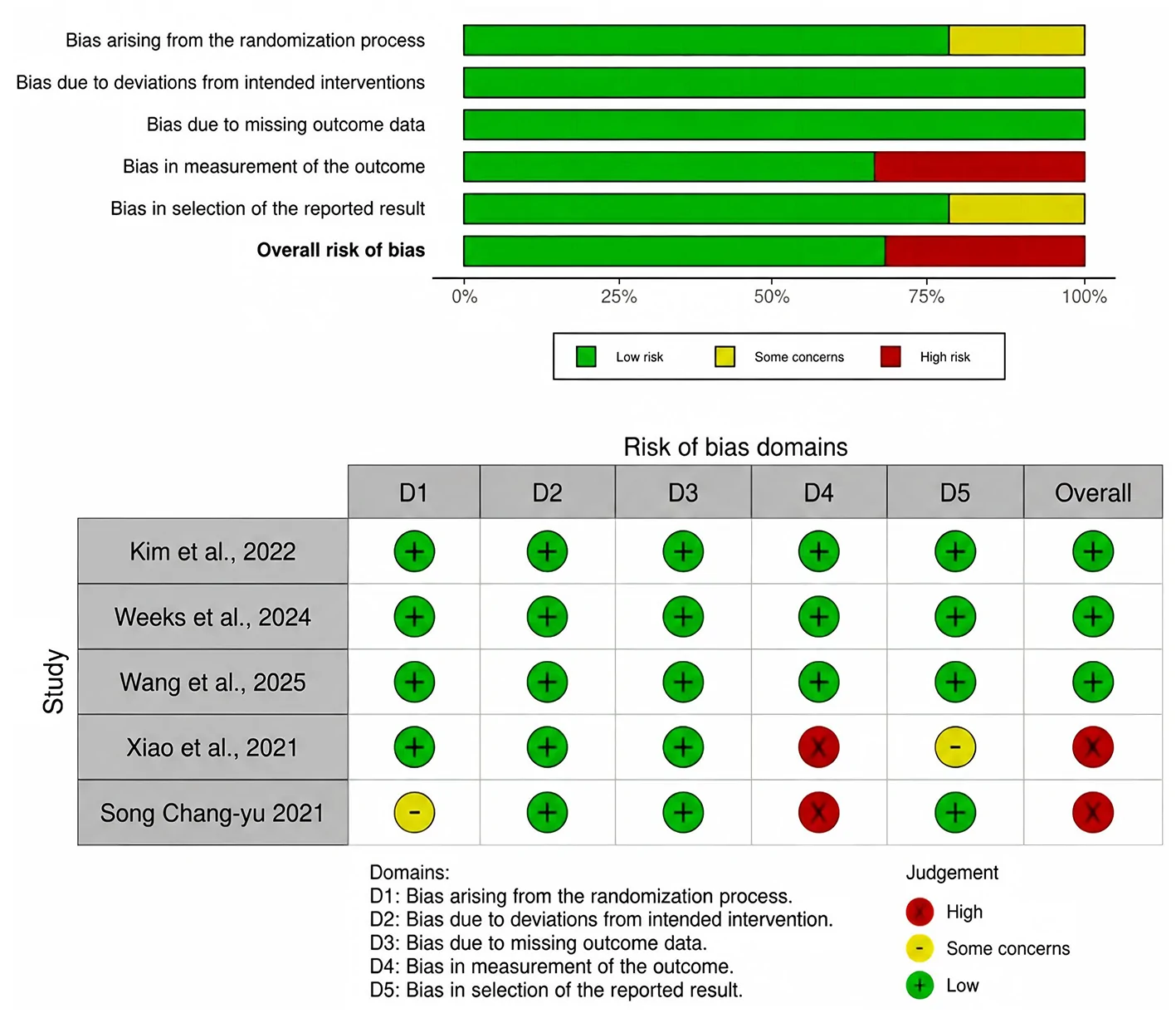
Risk of bias assessment of the included randomized controlled trials.

Based on the RoB 2 assessment, three trials were judged to be at low overall risk of bias and two at high overall risk. Domain-level and overall judgments are shown in Figure 2.

### 3.5 Quantitative synthesis of sleep outcomes

Quantitative synthesis was feasible for WASO and subjective sleep quality, because sufficiently comparable data with extractable variance were available. Other sleep-related outcomes were summarized narratively because reporting was limited or heterogeneous.

### 3.6 Wake after sleep onset (WASO)

**Figure 3:**
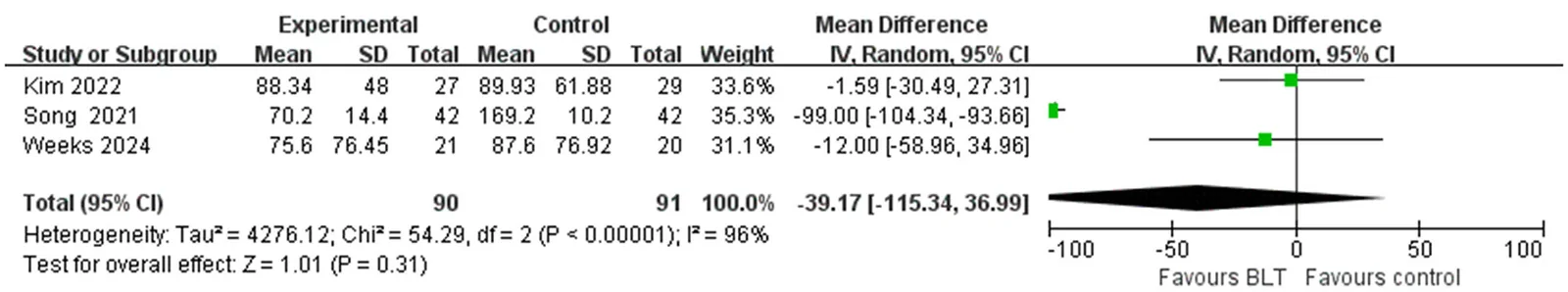
Forest plot of the meta-analysis of the effect of bright light therapy on wake after sleep onset (WASO) after stroke.

Three randomized controlled trials involving 181 participants reported WASO in min (16, 17, 25). The random-effects meta-analysis showed no statistically significant difference between the light-intervention and control groups, although the direction of effect favored light therapy (MD = −39.17 min, 95% CI −115.34 to 36.99; *P* = 0.31). Substantial heterogeneity was observed *(*χ^2^ = 54.29, df = 2, *P* < 0.001; *I*^2^ = 96%) (Figure 3).

**Figure 4:**
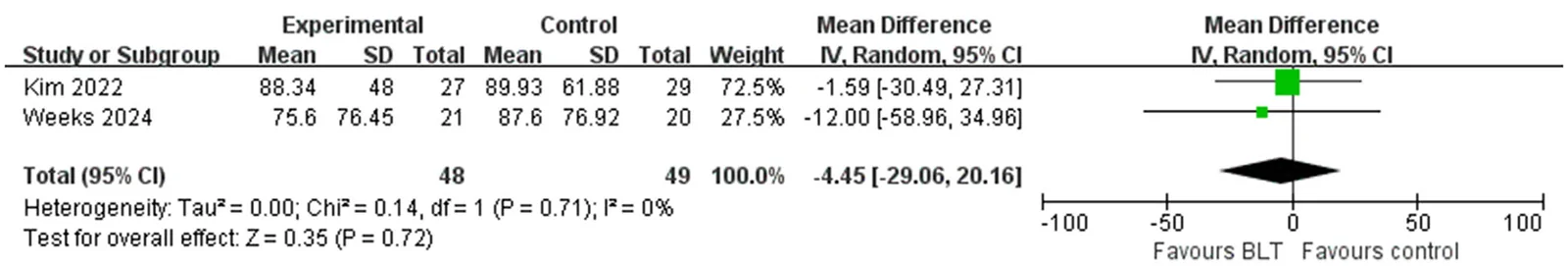
Sensitivity analysis of the effect of bright light therapy on wake after sleep onset (WASO).

In the *post-hoc* leave-one-out analysis, exclusion of Kim et al. (16) or Weeks et al. (17) did not materially reduce heterogeneity. After exclusion of Song (25) heterogeneity decreased to 0%, and the pooled effect estimate was reduced in magnitude to MD = −4.45 min (95% CI −29.06 to 20.16; *P* = 0.72), indicating that both the magnitude and heterogeneity of the pooled result were sensitive to the inclusion of this study (Figure 4).

### 3.7 Subjective sleep quality

**Figure 5:**
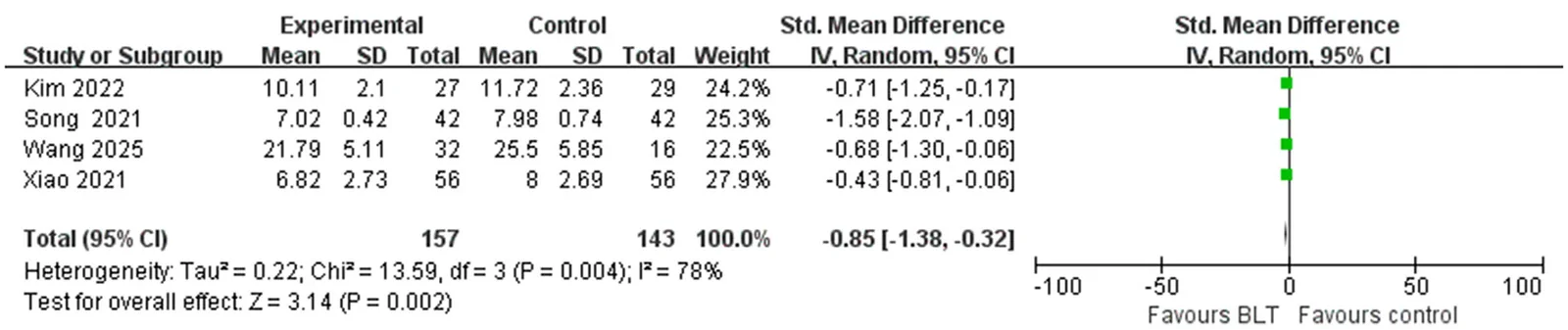
Forest plot of the effect of bright light therapy on subjective sleep quality in patients with post-stroke sleep disturbances.

Four randomized controlled trials involving 300 participants assessed subjective sleep quality using the PSQI or SRSS, with higher scores on both scales indicating poorer sleep (16, 23–25). The warm- and cold-light intervention arms in Wang et al. (23) were combined, with the shared control group included only once. The random-effects meta-analysis showed a statistically significant effect in favor of light therapy (SMD = −0.85, 95% CI −1.38 to −0.32; *P* = 0.002). Substantial heterogeneity was observed (χ^2^ = 13.59, df = 3, *P* = 0.004; *I*^2^ = 78%) (Figure 5).

In an analysis restricted to the three trials using the PSQI, (16, 24, 25) light therapy was associated with lower post-intervention PSQI scores (MD = −1.00 points, 95% CI −1.25 to −0.76; *P* < 0.001), with no evidence of statistical heterogeneity (χ^2^ = 1.26, df = 2, *P* = 0.53; *I*^2^ = 0%) (Supplementary Figure S1).

After exclusion of Song (25) study, the effect on subjective sleep quality remained statistically significant, although the pooled effect estimate was attenuated (SMD = −0.55, 95% CI −0.83 to −0.28; *P* < 0.001). No evidence of statistical heterogeneity was observed after exclusion of this study (χ^2^ = 0.88, df = 2, *P* = 0.64; *I*^2^ = 0%) (Supplementary Figure S2).

To assess the influence of the trial in which BLT was combined with escitalopram, an additional sensitivity analysis was conducted by excluding Xiao et al. (24). After exclusion of this trial, the effect of BLT on subjective sleep quality remained statistically significant (SMD = −1.01, 95% CI −1.61 to −0.40; *P* = 0.001), although substantial heterogeneity persisted (*I*^2^ = 73%) (Supplementary Figure S3). Thus, exclusion of the trial involving concomitant pharmacological treatment did not eliminate the statistically significant pooled effect.

### 3.8 Narrative synthesis of other sleep-related outcomes

Because of heterogeneity in outcome definitions, measurement methods, and reporting formats across the included trials, a narrative synthesis was undertaken for sleep-related outcomes that could not be quantitatively pooled.

### 3.9 Total sleep time (TST)

Total sleep time was reported in two trials but was not pooled because of heterogeneous reporting formats and incomplete variance data. Kim et al. (16) reported a higher post-intervention mean TST in the light-therapy group than in the control group (456.7 ± 159.0 min vs. 440.5 ± 121.6 min), whereas Song (25) reported a lower mean TST in the intervention group than in the control group (327.0 ± 13.8 min vs. 354.6 ± 7.2 min). Thus, the direction of the between-group difference in TST was inconsistent across the two trials.

### 3.10 Sleep efficiency (SE)

Sleep efficiency was reported in two trials using device-based sleep assessments. Quantitative pooling was not possible because variance data were unavailable for one study. Kim et al. (16) reported similar post-intervention mean SE values in the bright-light and filtered low-intensity placebo-light groups (73.2 ± 9.92% vs. 73.26 ± 9.72%). Song (25) reported higher post-intervention SE in the intervention group than in the control group (47.73% vs. 36.55%); however, standard deviations were not reported, precluding formal statistical comparison.

### 3.11 Overall qualitative interpretation

Across the included trials, the clearest statistically significant pooled finding was observed for subjective sleep quality, although heterogeneity was substantial. The pooled effect remained statistically significant after exclusion of Song (25), but the effect estimate was attenuated. By contrast, the pooled WASO effect was not statistically significant, and findings for TST and SE were inconsistent or not amenable to quantitative pooling.

## 4 Discussion

This systematic review and meta-analysis suggests that bright light therapy may improve subjective sleep quality in patients with post-stroke sleep disturbances. By contrast, evidence for objective sleep continuity remains uncertain, as the pooled WASO estimate was not statistically significant and was accompanied by substantial heterogeneity. Given that only five randomized controlled trials involving 343 participants were included, these findings should be considered preliminary. To our knowledge, this is the first quantitative synthesis to specifically evaluate the effects of light therapy on sleep outcomes after stroke; previous meta-analyses have focused primarily on primary insomnia (12), cancer-related sleep disturbances (26), or neurodegenerative disorders (27). Pharmacological treatments, including benzodiazepines and related hypnotics, remain commonly used for insomnia symptoms. However, their use may be limited by sedation, impaired balance, falls, cognitive effects, and the potential for dependence, particularly in older or neurologically vulnerable patients (11). These concerns provide a clinical rationale for investigating feasible non-pharmacological approaches such as light therapy. Because light therapy can be administered without substantial cognitive or motor demands, it may be particularly suitable for further evaluation during stroke rehabilitation (14).

### 4.1 Differential effects on subjective and objective sleep outcomes

The pooled analysis showed a statistically significant improvement in patient-reported sleep quality with light therapy, although heterogeneity was substantial (*I*^2^ = 78%). In the sensitivity analysis excluding Song (25), the effect remained statistically significant and heterogeneity decreased to 0%, although the pooled effect estimate was attenuated from an SMD of −0.85 to −0.55. The PSQI-specific analysis showed a similar direction of effect with no evidence of significant heterogeneity. Taken together, these findings indicate a consistent direction of effect for subjective sleep quality, but the magnitude of the pooled estimate varied across analyses. Further well-designed trials are therefore needed to determine the robustness and clinical relevance of this finding.

Findings for wake after sleep onset (WASO) were less conclusive than those for subjective sleep quality. Although the study-level estimates generally favored light therapy, the pooled effect was not statistically significant and showed substantial heterogeneity. Leave-one-out sensitivity analysis showed that exclusion of Song (25) reduced heterogeneity from 96% to 0% and attenuated the pooled mean difference from −39.17 to −4.45 min. This marked change indicates that the pooled estimate was sensitive to the inclusion of this study. Song (25) reported a larger between-group difference and smaller standard deviations than Kim et al. (16) and Weeks et al. (17). Methodological differences may also have contributed to the observed heterogeneity. Song (25) used cardiopulmonary coupling-based sleep monitoring and routine care as the comparator, whereas Kim et al. (16) and Weeks et al. (17) used wrist actigraphy and placebo-light comparators. However, the extent to which these differences contributed to the heterogeneity could not be determined because only three studies were available for quantitative synthesis.

Findings for total sleep time (TST) and sleep efficiency (SE) were also inconsistent across studies, with some trials showing differences in favor of light therapy and others showing minimal or opposite between-group differences. Taken together, the available evidence more consistently suggests an effect of BLT on subjective sleep quality than on objective sleep duration or efficiency. This pattern is broadly consistent with previous meta-analytic findings in cancer-related sleep disturbance (26) and chronic insomnia (12), in which light-based interventions have also been associated with improvements in patient-reported sleep outcomes. Overall, evidence regarding the effect of light therapy on objective sleep continuity remains inconclusive and requires confirmation in larger, methodologically standardized randomized trials.

### 4.2 Light parameters and potential circadian mechanisms

The included interventions differed considerably in illuminance, spectral composition, exposure duration, intervention period, and light-delivery device. Nevertheless, all interventions were administered in the morning or shortly after awakening, consistent with the timing-dependent nature of circadian responses to light. Photopic illuminance alone cannot fully characterize the non-visual biological effects of light, because these effects are also shaped by spectral composition and melanopsin-mediated photoreception (21). Evidence from other populations indirectly supports the relevance of spectral characteristics. In patients with seasonal affective disorder, low-illuminance blue-enriched white light produced therapeutic effects comparable to those of standard 10,000-lx white light (28), whereas in healthy adults, blue light elicited stronger brain responses than violet or green light under matched photon conditions (29). However, the extent to which these spectrum-dependent effects apply to individuals with post-stroke sleep disturbances remains uncertain and requires further investigation in stroke-specific populations.

Circadian modulation nevertheless provides a biologically plausible mechanism that may underlie the observed improvement in subjective sleep quality. Stroke may disrupt sleep-wake regulation and alter circadian timing, melatonin and cortisol secretion, core body temperature, and rest-activity rhythms (30–32). Light signals detected by rods, cones, and melanopsin-containing intrinsically photosensitive retinal ganglion cells are transmitted via the retinohypothalamic tract to the suprachiasmatic nucleus, thereby influencing circadian timing and melatonin regulation (21, 33). Morning light exposure may therefore help reinforce sleep–wake organization.

A previous study in institutionalized older adults reported improvements in circadian rhythm parameters and sleep quality following morning bright light exposure (34), providing supportive evidence for a potential role of morning light in sleep-wake regulation. Whether similar circadian-mediated effects contribute to sleep improvement after stroke remains to be determined.

However, the included trials primarily assessed sleep-related outcomes, including subjective sleep quality, sleep duration, and sleep continuity, rather than direct measures of circadian timing or rhythmicity. Only Wang et al. (23) measured serum melatonin at a single standardized morning time point, but a single morning measurement was insufficient to characterize dim-light melatonin onset, peak timing, rhythm amplitude, or the full circadian secretion profile. No trial reported established actigraphy-derived circadian indices such as L5, M10, relative amplitude, interdaily stability, or intradaily variability. Moreover, none provided group-specific numerical values for melanopic equivalent daylight illuminance (melanopic EDI) or melanopic irradiance. Collectively, the available evidence is consistent with a potential role of circadian regulation in the effects of light therapy; however, direct mechanistic assessments are still needed to determine how circadian pathways contribute to sleep improvement after stroke. Beyond stroke, light therapy has also been investigated in other acquired brain injury populations using designs informed by circadian principles. For example, a randomized controlled trial in individuals recovering from mild traumatic brain injury investigated morning blue-wavelength light exposure using standardized intervention timing and actigraphy-based assessment of sleep timing and circadian-related sleep-wake changes (35). These studies provide useful methodological insights for future post-stroke light therapy trials, particularly regarding intervention timing and the incorporation of objective circadian-related outcomes.

### 4.3 Clinical implications

From a clinical perspective, light therapy may represent a feasible non-pharmacological approach to managing sleep disturbances after stroke. Morning light exposure is relatively brief, can be incorporated into routine rehabilitation schedules, and requires relatively little cognitive or physical engagement, which may be advantageous for stroke survivors with functional limitations or those who have difficulty participating in more complex behavioral sleep interventions.

The present findings suggest that light therapy may be particularly relevant to improving patient-reported sleep quality, although the magnitude of effects and its clinical applicability require further evaluation. These findings provide a rationale for further investigation of light therapy as an individualized component of sleep management during stroke rehabilitation. Future studies should focus on optimizing intervention parameters and implementation strategies, including the timing, illuminance, spectral characteristics, duration, and delivery modality of light exposure, to identify the most appropriate light-therapy protocols for patients with post-stroke sleep disturbances.

### 4.4 Strengths and limitations

This review has several strengths. To our knowledge, it is the first quantitative synthesis specifically evaluating the effects of light therapy on sleep outcomes after stroke. The review included both English- and Chinese-language databases, assessed both subjective and objective sleep outcomes, systematically characterized available light-intervention parameters, and conducted sensitivity analyses to evaluate the robustness of the pooled findings and potential sources of heterogeneity.

Several limitations should also be acknowledged. First, only five randomized controlled trials were included, and individual trial sample sizes were generally small, limiting the precision and certainty of the pooled estimates. Second, considerable between-study variability was present in participant characteristics, sleep phenotypes, light parameters, intervention duration, comparator conditions, and sleep-assessment methods. Third, incomplete reporting of variance data prevented quantitative synthesis of several objective sleep outcomes. Fourth, the pooled WASO estimate and its heterogeneity were sensitive to the inclusion of one study, and the limited number of trials precluded meaningful subgroup analyses according to light intensity, intervention timing, or rehabilitation setting. Fifth, direct circadian outcomes and biologically relevant light-dose metrics, including melanopic measures, were limited or unavailable. Finally, most interventions involved relatively short treatment periods, and long-term effects remain uncertain.

## 5 Conclusion

Bright light therapy may improve subjective sleep quality in individuals with post-stroke sleep disturbances and warrants further evaluation as a feasible non-pharmacological approach during stroke rehabilitation. However, evidence regarding its effects on objective sleep continuity remains uncertain, with substantial heterogeneity across studies. Larger, well-designed randomized controlled trials using standardized light-intervention protocols, comprehensive sleep assessments, and direct circadian measures are needed to better define the clinical effects and underlying mechanisms of light therapy after stroke.

## Data Availability

The original contributions presented in the study are included in the article/Supplementary material, further inquiries can be directed to the corresponding authors.
