# Supplementary material for: Bright light therapy for post-stroke sleep disturbances: a systematic review and meta-analysis
Source: Front Neurol. 2026 Sep 7;17:1875347. doi: 10.3389/fneur.2026.1875347 (PMC13591042; doi:10.3389/fneur.2026.1875347)
Supplement: Supplementary file 1 [file Data_Sheet_1.PDF]

Search Strategies
A comprehensive and systematic literature search was conducted in Chinese and international electronic databases from database inception to April 9, 2026, without restrictions on publication date. The search strategy combined controlled vocabulary terms and free-text terms related to bright light therapy, stroke, and sleep disturbances. Study design was assessed during title/abstract and full-text screening according to the predefined eligibility criteria. Searches were limited to articles published in English or Chinese. The detailed search strategies for each database are presented below.

Chinese Databases
China National Knowledge Infrastructure (CNKI)
SU=(光照疗法+强光疗法+明亮光照+光暴露+日光暴露+光疗+光照治疗) And SU=(脑卒中+卒中+脑梗死+脑梗塞+缺血性脑卒中+脑出血+出血性脑卒中+脑缺血)And SU=(睡眠障碍 OR 失眠 OR 睡眠-觉醒障碍 OR 昼夜节律 OR 昼夜节律失调 OR 睡眠-觉醒节律紊乱)
Results: 11 record

Wanfang Data
主题:(光照疗法 OR 强光疗法 OR 明亮光照 OR 光暴露 OR 日光暴露 OR 光疗 OR 光照治疗) AND 主题:(脑卒中 OR 卒中 OR 脑梗死 OR 脑梗塞 OR 缺血性脑卒中 OR 脑出血 OR 出血性脑卒中 OR 脑缺血) AND 主题:(睡眠障碍 OR 失眠 OR 睡眠-觉醒障碍 OR 昼夜节律 OR 昼夜节律失调 OR 睡眠-觉醒节律紊乱)
Results: 34 records

VIP Database
M=("光照疗法" OR "亮光疗法" OR "强光疗法" OR "明亮光照") AND M=(脑卒中 OR 卒中 OR 中风 OR 脑梗死 OR 脑梗塞 OR 缺血性脑卒中 OR 脑出血 OR 出血性脑卒中 OR 脑缺血) AND M=(睡眠障碍 OR 失眠 OR 睡眠-觉醒障碍 OR 昼夜节律 OR 昼夜节律失调 OR 睡眠-觉醒节律紊乱)
Results: 7 records

SinoMed (Chinese Biomedical Literature Database)
(光照疗法 OR 明亮光照 OR 强光疗法 OR 日光照射 OR 光照干预 OR 光照治疗) AND (脑卒中 OR 卒中 OR 中风 OR 脑梗死 OR 脑梗塞 OR 缺血性脑卒中 OR 脑出血 OR 出血性脑卒中 OR 脑缺血) AND (睡眠障碍 OR 失眠 OR 睡眠-觉醒障碍 OR 昼夜节律 OR 昼夜节律失调 OR 睡眠-觉醒节律紊乱)
Results: 4 records

International Databases
PubMed
("Phototherapy"[Mesh] OR phototherapy[tiab] OR "light therapy"[tiab] OR "bright light"[tiab] OR "bright light therapy"[tiab] OR "light exposure"[tiab] OR "daylight exposure"[tiab]) AND ("Stroke"[Mesh] OR "Cerebral Hemorrhage"[Mesh] OR "Brain Ischemia"[Mesh] OR stroke[tiab] OR "ischemic stroke"[tiab] OR "cerebral infarction"[tiab] OR "brain infarction"[tiab] OR "cerebral hemorrhage"[tiab] OR "intracerebral hemorrhage"[tiab] OR "post-stroke"[tiab]) AND ("Sleep Wake Disorders"[Mesh] OR "Sleep Initiation and Maintenance Disorders"[Mesh] OR insomnia[tiab] OR "sleep disorder*"[tiab] OR "sleep disturbance*"[tiab] OR "sleep-wake"[tiab] OR "circadian rhythm"[tiab] OR "circadian rhythm sleep disorder*"[tiab] OR "sleep quality"[tiab] OR "Pittsburgh Sleep Quality Index"[tiab] OR PSQI[tiab])
Results: 29 records
Web of Science

TS=("light therapy" OR "bright light" OR "bright light therapy" OR "light exposure" OR "daylight exposure" OR phototherapy) AND TS=(stroke OR "ischemic stroke" OR "cerebral infarction" OR "brain infarction" OR "cerebral hemorrhage" OR "intracerebral hemorrhage") AND TS=(insomnia OR "sleep disorder*" OR "sleep disturbance*" OR "sleep quality" OR "sleep-wake" OR "circadian rhythm" OR "circadian rhythm sleep disorder*" OR "Pittsburgh Sleep Quality Index" OR PSQI)
Results: 30 records

Embase (via Elsevier)
('phototherapy'/exp OR 'phototherapy':ti,ab,kw OR 'light therapy':ti,ab,kw OR 'bright light':ti,ab,kw OR 'bright light therapy':ti,ab,kw OR 'light exposure':ti,ab,kw OR 'daylight exposure':ti,ab,kw) AND ('stroke'/exp OR 'brain ischemia'/exp OR 'cerebral hemorrhage'/exp OR stroke:ti,ab,kw OR 'ischemic stroke':ti,ab,kw OR 'cerebral infarction':ti,ab,kw OR 'brain infarction':ti,ab,kw OR 'cerebral hemorrhage':ti,ab,kw OR 'intracerebral hemorrhage':ti,ab,kw) AND ('sleep disorder'/exp OR insomnia:ti,ab,kw OR 'sleep disorder*':ti,ab,kw OR 'sleep disturbance*':ti,ab,kw OR 'sleep quality':ti,ab,kw OR 'sleep-wake':ti,ab,kw OR 'circadian rhythm':ti,ab,kw OR 'circadian rhythm sleep disorder*':ti,ab,kw OR 'pittsburgh sleep quality index':ti,ab,kw OR psqi:ti,ab,kw) AND ([chinese]/lim OR [english]/lim)
Results: 128 records

Cochrane Library
("light therapy" OR "bright light" OR "bright light therapy" OR "light exposure" OR "daylight exposure" OR phototherapy):ti,ab,kw AND (sleep disorder* OR insomnia OR "sleep quality" OR sleep disturbance* OR "sleep-wake" OR "circadian rhythm" OR circadian NEXT rhythm NEXT sleep NEXT disorder* OR "Pittsburgh Sleep Quality Index" OR psqi):ti,ab,kw AND (stroke OR "cerebral infarction" OR "brain infarction" OR "ischemic stroke" OR "intracerebral hemorrhage" OR "cerebral hemorrhage"):ti,ab,kw
Results: 31 record

Scopus
TITLE-ABS-KEY("light therapy" OR "bright light" OR "bright light therapy" OR "light exposure" OR "daylight exposure" OR phototherapy) AND TITLE-ABS-KEY(stroke OR "ischemic stroke" OR "cerebral infarction" OR "brain infarction" OR "cerebral hemorrhage" OR "intracerebral hemorrhage") AND TITLE-ABS-KEY(insomnia OR "sleep disorder*" OR "sleep disturbance*" OR "sleep quality" OR "sleep-wake" OR "circadian rhythm" OR "circadian rhythm sleep disorder*" OR "Pittsburgh Sleep Quality Index" OR psqi)
Results: 66 records

EBSCOhost multi-database search
Databases contributing records: MEDLINE, MEDLINE Complete, CINAHL Plus with Full Text, APA PsycInfo, Academic Search Premier, and The Belt and Road Initiative Reference Source.
("light therapy" OR "bright light" OR "bright light therapy" OR "light exposure" OR "daylight exposure" OR phototherapy) AND (stroke OR "ischemic stroke" OR "cerebral infarction" OR "brain infarction" OR "cerebral hemorrhage" OR "intracerebral hemorrhage") AND (insomnia OR "sleep disorder*" OR "sleep disturbance*" OR "sleep quality" OR "sleep-wake" OR "circadian rhythm" OR "circadian rhythm sleep disorder*" OR "Pittsburgh Sleep Quality Index" OR PSQI)
Results: 46 records

Summary of Records Identified
A total of 386 records were identified through database searching across all sources. After removal of 133 duplicate records, 253 records remained for title and abstract screening. The study selection process is illustrated in Figure 1 (PRISMA flow diagram).
