# Supplementary material for: Bright light therapy for post-stroke sleep disturbances: a systematic review and meta-analysis
Source: Front Neurol. 2026 Sep 7;17:1875347. doi: 10.3389/fneur.2026.1875347 (PMC13591042; doi:10.3389/fneur.2026.1875347)
Supplement: Supplementary file 2 [file Data_Sheet_2.PDF]

Study or Subgroup	Experimental Mean	Experimental SD	Experimental Total	Control Mean	Control SD	Control Total	Weight	Mean Difference IV, Random, 95% CI
Kim 2022	10.11	2.1	27	11.72	2.36	29	4.4%	-1.61 [-2.78, -0.44]
Song 2021	7.02	0.42	42	7.98	0.74	42	89.7%	-0.96 [-1.22, -0.70]
Xiao 2021	6.82	2.73	56	8	2.69	56	5.9%	-1.18 [-2.18, -0.18]
**Total (95% CI)**			**125**			**127**	**100.0%**	**-1.00 [-1.25, -0.76]**

Heterogeneity: Tau² = 0.00; Chi² = 1.26, df = 2 (P = 0.53); I² = 0%
Test for overall effect: Z = 8.05 (P < 0.00001)

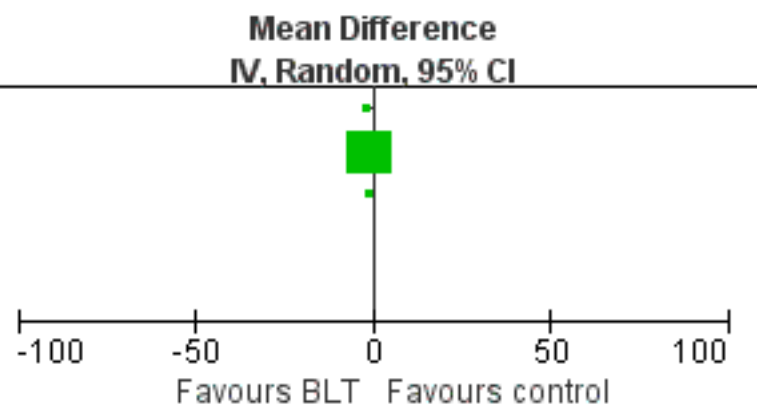

Supplementary Figure S1. Forest plot of the effect of bright light therapy on Pittsburgh Sleep Quality Index (PSQI) scores in patients with post-stroke sleep disturbances.

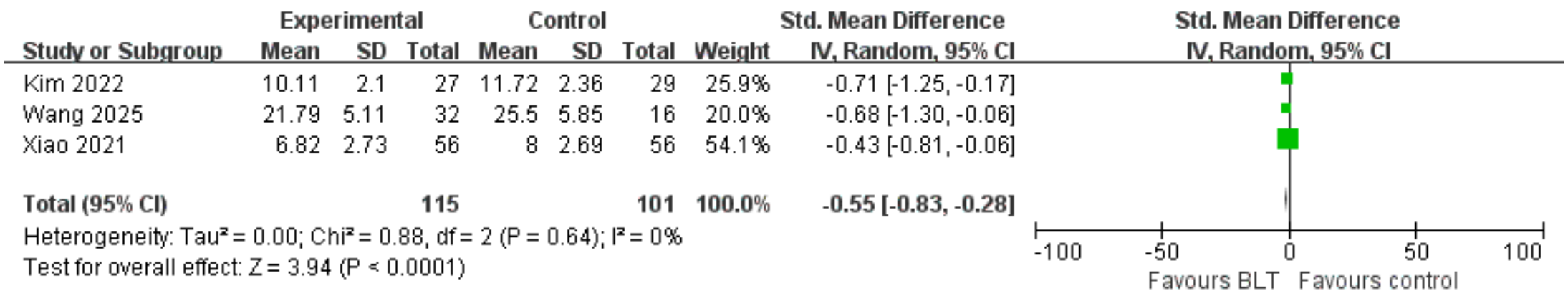

Supplementary Figure S2. Sensitivity analysis of the effect of bright light therapy on subjective sleep quality after excluding Song Chang-yu (2021).

	Experimental			Control				Std. Mean Difference
Study or Subgroup	Mean	SD	Total	Mean	SD	Total	Weight	IV, Random, 95% CI
Kim 2022	10.11	2.1	27	11.72	2.36	29	33.6%	-0.71 [-1.25, -0.17]
Song 2021	7.02	0.42	42	7.98	0.74	42	35.2%	-1.58 [-2.07, -1.09]
Wang 2025	21.79	5.11	32	25.5	5.85	16	31.1%	-0.68 [-1.30, -0.06]
**Total (95% CI)**			**101**			**87**	**100.0%**	**-1.01 [-1.61, -0.40]**

Heterogeneity: $Tau^2 = 0.21$; $Chi^2 = 7.36$, $df = 2$ ($P = 0.03$); $I^2 = 73\%$

Test for overall effect: $Z = 3.25$ ($P = 0.001$)

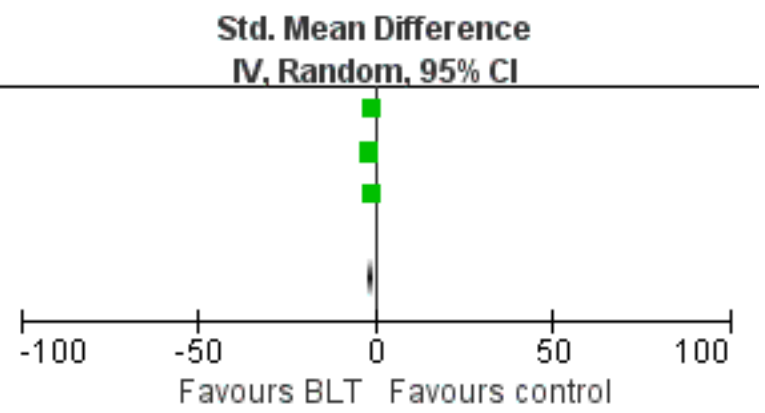

Supplementary Figure S3. Sensitivity analysis of the effect of bright light therapy on subjective sleep quality after excluding Xiao et al. (2021).
